# The association analysis between *CYP24A1* genetic polymorphisms and the risk of ischemic stroke in Chinese Han population

**DOI:** 10.1002/brb3.1503

**Published:** 2019-12-24

**Authors:** Wei Yang, Fenghui Ma, Li Wang, Xue He, Hengxun Zhang, Jianwen Zheng, Yuhe Wang, Tianbo Jin, Dongya Yuan, Yongjun He

**Affiliations:** ^1^ Key Laboratory of Molecular Mechanism and Intervention Research for Plateau Diseases of Tibet Autonomous Region School of Medicine Xizang Minzu University Xianyang China; ^2^ Department of Emergency the Affiliated Hospital of Xizang Minzu University Xianyang China; ^3^ Medical Examination Center Tangdu Hospital the Fourth Military Medical University Xi’an China; ^4^ School of Basic Medical Science Xizang Minzu University Xianyang China; ^5^ Department of Neurology the Affiliated Hospital of Xizang Minzu University Xianyang China; ^6^ Department of Clinical Laboratory the Affiliated Hospital of Xizang Minzu University Xianyang China; ^7^ Key Laboratory of Resource Biology and Biotechnology in Western China (Northwest University) Ministry of Education Northwest University Xi’an China

**Keywords:** *CYP24A1*, genetic polymorphisms, ischemic stroke

## Abstract

**Aims:**

Stroke is a complicated neurological disease and the second leading cause of death in the world. We aimed to investigate the association between *CYP24A1* genetic polymorphisms and ischemic stroke risk.

**Methods:**

In this case–control study, four single‐nucleotide polymorphisms of *CYP24A1* were selected and genotyped by MassARRAY platform in Chinese Han population. Odds ratios and 95% confidence intervals were calculated via logistic regression analysis with adjustment in genetic models.

**Results:**

Our results indicated that *CYP24A1* variant (rs1570669) was associated with the decreased risk of ischemic stroke (OR = 0.60, *p* < .001). Stratification analysis showed that the rs6068816 could enhance the ischemic stroke risk by 1.64 times (OR = 1.64, *p* = .028), while rs1570669 played protective role (OR = 0.63, *p* = .044) in age >64 years. The rs2762934 had an increased ischemic stroke susceptibility (OR = 1.62, *p* = .033); however, rs1570669 might reduce stroke risk (OR = 0.61, *p* = .015) in age ≤64 years. The rs1570669 depressed ischemic stroke susceptibility both in female and male patients (OR = 0.46, *p* = .002; OR = 0.69, *p* = .033, respectively), and rs2296241 would weaken the risk in male (OR = 0.63, *p* = .012). The rs1570669 was associated with decreased risk of ischemic stroke with hypertension (OR = 0.56, *p* = .042).

**Conclusion:**

Our study gave the evidences that *CYP24A1* genetic polymorphisms were significantly associated with ischemic stroke patients, which would provide useful information of assessment or possible diagnostic markers for ischemic stroke.

## Graphical abstract

The results indicated that CYP24A1 rs1570669 was significantly association with stroke risk. Stratification analysis found that rs6068816 could enhance the stroke risk by 1.64 times at age >64 years, and rs2762934 had an increased stroke susceptibility in age ≤64 years. We observed that CYP24A1 rs2296241was associated with the increased stroke risk in males than in females.

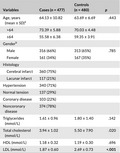

## 1 INTRODUCTION

Stroke, also named as brain attack, is a complex neurological disease and the second most common cause of death in the world (Katan & Luft, 2018; Naghavi et al., 2017). With the characteristics of high incidence rate, high disability rate, high death rate, high recurrence rate and more complications, it gives to a serious hazard to human health and safety (Gorelick, 2019). Stroke is divided into ischemic stroke (85% of all strokes) and hemorrhagic stroke (15% of all stroke). It occurs when blood in a certain area of the brain is interrupted or reduced, usually caused by a thrombus or a rupture or leakage of blood vessels. There are many risk factors for the development of stroke, such as race, sex, age, hypertension, diabetes mellitus, obesity, atherosclerosis, and dyslipidemia (Bhat et al., 2008; Hankey, 2003; Khoury et al., 2013; Kleindorfer et al., 2010; Tirschwell et al., 2004). In addition, genetic factor was also known to be a very important risk factor for stroke (Boehme, Esenwa, & Elkind, 2017). Polymorphisms of some genes have been identified to associate with the development of stroke, including *HTRA1* (Menezes Cordeiro et al., 2015), *ABO* (Williams et al., 2013), *FOXF2* (Chauhan et al., 2016), and *PITX2* (Gretarsdottir et al., 2008).

*CYP24A1* gene, cytochrome P450 24 subfamily A member 1, encodes a 24‐hydroxylase, which can degrade the active form of vitamin D, 1, 25‐dihydroxyvitamin D through multiple pathways (Blunt, DeLuca, & Schnoes, 1968; Jones, Prosser, & Kaufmann, 2012, 2014). It has been found that the functional deletion of *CYP24A1* gene leads to an increased concentration of 1, 25‐dihydroxyvitamin D in serum (Jones et al., 2012; Roff & Wilson, 2008). And many studies have indicated that vitamin D deficiency is a risk and serious factor for stroke development (Anderson et al., 2010; Poole, Nigel, et al., 2006). Therefore, they believe that the single‐nucleotide polymorphisms (SNPs) in enzymes involved in vitamin D metabolism such as *CYP24A1* gene is closely related to the occurrence of stroke. However, there is very limited information about the polymorphism of the *CYP24A1* gene association with stroke risk. Adali's research showed that *CYP24A1* (rs927650) was associated with susceptibility of stroke with hypertensive, smoker, diabetic, and obese individuals (Turkanoglu Ozcelik et al., 2018). These studies suggest that *CYP24A1* genetic polymorphism may be associated with stroke.

In this study, *CYP24A1* SNPs effecting on ischemic stroke patients susceptibility were assessed in the Chinese Han population with case–control method. We also investigated the other risk factors (age, gender, hypertension, coronary disease) of ischemic stroke related to polymorphism of *CYP24A1* gene. Our present study will give available information for prevention and management of ischemic stroke.

## 2 MATERIALS AND METHODS

### 2.1 Study subjects

All subject populations in this case–control study were recruited from the Affiliated Hospital of Xizang Minzu University including 477 unrelated ischemic stroke patients and 480 healthy individuals. The volunteers involved in our study were informed the intention of the sample collection and obtained written informed consent from each participant. Cases were selected from patients with ischemic stroke who were firstly diagnosed by neurological examination and brain computer tomography (CT) scan according to the clinical practice guideline for stroke in the World Health Organization. The inclusion criteria of patients are having anterior circulation stroke. In addition, the patients must meet the following exclusion criteria: the initial diagnosis is not stroke; caused by brain tumors, brain injuries, and hematological diseases; having family history of stroke; having history of autoimmune diseases, endocrine system, neoplasms, osteoarthrosis, and mental illness. Health controls without history of stroke or transient ischemic attack and other diseases were randomly chosen from unrelated Chinese Han population who had a physical examination in the same hospital. Our study was approved by the ethics committee of the Affiliated Hospital of Xizang Minzu University. All experiments were carried out depending on the guideline of Helsinki's Declaration.

### 2.2 SNP selection and genotyping

Four SNPs (rs2762934, rs1570669, rs6068816, and rs2296241) in *CYP24A1* gene were selected from the databases of the 1,000 Genomes Project with a minor allele frequency ≥0.05 in our present study. The genomic DNA of peripheral blood samples in all subjects was extracted with a blood genomic DNA purification kit (GoldMag) following the manufacturer's protocol. The purity and concentration of genomic DNA were detected by NanoDrop 2000C spectrophotometer (Thermo Scientific; Gabriel et al., 2009) and then kept at −20°C before further analysis. Primers for PCR amplification were designed using the Agena Bioscience Assay Design Suite V2.0 software (https://agenacx.com/online-tools/). SNP genotyping was identified by Agena MassARRAY iPLEX platform, and the data management and analysis were carried out using Agena Bioscience TYPER version 4.0 software.

### 2.3 Statistical analysis

The differences of demographic characteristics in case and control subjects were analyzed by independent sample Student's *t* test for continuous variables and Pearson's χ^2^ tests for categorical variables. Hardy–Weinberg equilibrium (HWE) was used to assess the genotype frequencies of control group with the Chi‐squared test. The comparisons of the distribution in SNP allele and genotype frequencies between case and control were checked by a Pearson Chi‐squared test or Fisher's exact test. Odds ratios (ORs) and 95% confidence intervals (CIs) were calculated to evaluate the associations between *CYP24A1* SNPs and ischemic stroke risk using logistic regression analysis with or without adjustment of age and gender. Multiple inheritance models were used for testing the relationship between SNPs and ischemic stroke risk using logistic regression analysis with adjustments by PLINK software. In addition, the stratified analyses were also carried out to access the association between SNPs and ischemic stroke risk in different subgroups (age, gender, and other risk factors). All statistical analyses were calculated with SPSS v17.0 (SPSS), and a two‐tailed *p* < .05 was considered to be statistically significant.

## 3 RESULTS

### 3.1 Characteristics of study individuals

A total number of 957 subjects took part in our present study, including 477 ischemic stroke patients (316 males, 161 females, averaged age 64.13 ± 10.82) and 480 healthy controls (313 males, 167 females, averaged age 63.69 ± 6.69). There were no significant differences in age (*p* = .433) and gender (*p* = .785) between the cases and controls. The detail characteristic information was presented in Table 1.

**Table 1:** Basic characteristic of individuals in this study

Variables	Cases (*n* = 477)	Controls (*n* = 480)	*p*
Age, years (mean ± *SD*)^a^	64.13 ± 10.82	63.69 ± 6.69	.443
>64	73.39 ± 5.88	70.03 ± 4.48	
≤64	55.58 ± 6.38	59.35 ± 3.91	
Gender^b^			
Male	316 (66%)	313 (65%)	.785
Female	161 (34%)	167 (35%)	
Histology			
Cerebral infarct	360 (75%)		0.645
Lacunar infarct	117 (21%)		
Hypertension	340 (71%)		0.362
Normal tension	137 (29%)		
Coronary disease	103 (22%)		0.020
Noncoronary disease	374 (78%)		
Triglycerides (mmol/L)	1.61 ± 0.96	1.80 ± 1.40	.142
Total cholesterol (mmol/L)	3.94 ± 1.02	5.50 ± 7.90	.020
HDL (mmol/L)	1.18 ± 0.32	1.19 ± 0.30	.696
LDL (mmol/L)	1.87 ± 0.60	2.69 ± 0.73	**<.001**

Note

*p* < .05 indicates statistical significance.

Abbreviations: HDL, high‐density lipoprotein; LDL, low‐density lipoprotein.

a Student's *t* test is used.

b Pearson's χ^2^ test is used.

### 3.2 Association between *CYP24A1* polymorphisms and ischemic stroke risk assessment

Four SNPs of *CYP24A1* gene were analyzed in our present study. The distribution of allele frequencies and basic information of all SNPs were listed in Table 2. All the candidate SNPs conformed to the HWE (*p* > .05) which indicated appropriate SNP selection. And all allele frequencies of *CYP24A1* SNPs were not significantly associated with ischemic stroke risk (*p* > .05). We examined the association between *CYP24A1* SNPs genotypes and ischemic stroke risk in genetic models by logistic regression analysis with adjustments for age and gender, as shown in Table 3. Our results showed that the rs1570669 AG genotype in *CYP24A1* was associated with a decreased risk of ischemic stroke (OR = 0.60, 95% CI = 0.46–0.80, *p* < .001). The AG–AA genotype of rs1570669 in *CYP24A1* gene also decreased the risk of ischemic stroke (OR = 0.68, 95% CI = 0.52–0.88, *p* = .003).

**Table 2:** The distribution of allele frequencies of *CYP24A1* SNPs in case and control

SNP ID	Alleles (minor/major)	MAF		O (HET)	E (HET)	*p* a‐HWE	OR (95% CI)	*p* b
		Case	Control					
rs2762934	A/G	0.103	0.097	0.189	0.195	.487	1.07 (0.81–1.42)	.630
rs1570669	A/G	0.228	0.238	0.472	0.475	.923	0.86 (0.71–1.03)	.102
rs6068816	T/C	0.229	0.221	0.437	0.442	.837	1.12 (0.93–1.36)	.234
rs2296241	A/G	0.245	0.248	0.530	0.496	.142	0.91 (0.76–1.09)	.285

Abbreviations: E (HET), expected heterozygosity; HWE, Hardy–Weinberg equilibrium; MAF, minor allele frequency; O (HET), observed heterozygosity; SNP, single‐nucleotide polymorphisms.

a *p* < .05 are excluded;

b *p* values were calculated by two‐sided χ^2^, *p^b^* < .05 indicates statistical significance.

**Table 3:** The SNPs of *CYP24A1* gene association with ischemic stroke risk

SNP	Genotype	Case	Control	Adjusted analysis		Crude analysis	
				OR (95% CI)	*p* a	OR (95% CI)	*p* b
rs2762934	GG	370	382	1.00		1.00	
	AG	103	91	1.17 (0.85–1.60)	.333	1.17 (0.85–1.60)	.334
	AA	4	7	0.58 (0.17–2.02)	.395	0.59 (0.17–2.03)	.403
	AG–AA	107	98	1.13 (0.83–1.54)	.449	1.13 (0.83–1.54)	.448
rs1570669	GG	224	180	1.00		1.00	
	AG	170	226	0.60 (0.46–0.80)	**<.001**	0.60 (0.46–0.80)	**<.001**
	AA	83	73	1.40 (0.63–3.10)	.628	0.91 (0.63–1.32)	.633
	AG–AA	253	299	0.68 (0.52–0.88)	**.003**	0.68 (0.53–0.88)	**.003**
rs6068816	CC	190	216	1.00		1.00	
	TC	235	209	1.27 (0.97–1.67)	.083	1.28 (0.98–1.67)	.074
	TT	52	53	1.11 (0.72–1.70)	.643	1.12 (0.73–1.72)	.618
	TC–TT	287	262	1.24 (0.96–1.60)	.105	1.25 (0.96–1.61)	.094
rs2296241	GG	156	133	1.00		1.00	
	AG	229	254	0.77 (0.57–1.03)	.080	0.77 (0.57–1.03)	.078
	AA	92	92	0.86 (0.59–1.25)	.423	0.85 (0.59–1.23)	.398
	AG–AA	321	346	0.79 (0.60–1.05)	.102	0.79 (0.60–1.04)	.097

Abbreviations: CI, confidence interval; OR, odds ratio; SNP, single‐nucleotide polymorphism.

*p* a<0.05 indicates statistical significance.

a *p*‐values were calculated from unconditional logistic regression analysis.

b *p*‐values were calculated by unconditional logistic regression analysis with adjustments for age and gender.

### 3.3 Effects of *CYP24A1* polymorphisms on ischemic stroke susceptibility in different stratifications

The stratified analysis was performed to assess the influence of SNPs genotypes on ischemic stroke risk in multiple inheritance models. Because the average age of case and control group is 64 years old in our study populations, we stratified by age of 64 years. Association between SNPs genotypes and ischemic stroke risk in age stratification was shown in Table 4. In age >64 years, the rs1570669 in *CYP24A1* could decrease the risk of ischemic stroke (AG genotype, OR = 0.63, 95% CI = 0.40–0.99, *p* = .044), and also reduced the ischemic stroke risk (AG–AA genotype, OR = 0.66, 95% CI = 0.43–0.99, *p* = .044). Besides, the TC genotype of rs6068816 enhanced the ischemic stroke risk by 1.64 times in codominant model (OR = 1.64, 95% CI = 1.06–2.53, *p* = .028). In age ≤64 years, the rs2762934 would improve the ischemic stroke susceptibility (AG genotype, OR = 1.62, 95% CI = 1.04–2.52, *p* = .033; AG–AA genotype, OR = 1.62, 95% CI = 1.05–2.51, *p* = .031, respectively). The AG genotype of rs1570669 had a depressed correlation with ischemic stroke risk (OR = 0.61, 95% CI = 0.41–0.91, *p* = .015). Association analyses between SNPs genotypes and ischemic stroke risk in gender stratification were listed in Table 5. In females, the rs1570669 was related to a decreased ischemic stroke risk (AG genotype, OR = 0.46, 95% CI = 0.28–0.74, *p* = .002; AG–AA genotype, OR = 0.58, 95% CI = 0.37–0.91, *p* = .018, respectively). In males, the rs1570669 could reduce the occurrence of ischemic stroke (AG genotype, OR = 0.69, 95% CI = 0.49–0.97, *p* = .033; AG–AA genotype, OR = 0.73, 95% CI = 0.53–1.00, *p* = .049, respectively). And the AG genotype of rs2296241 had a depressed risk of ischemic stroke (OR = 0.63, 95% CI = 0.44–0.90, *p* = .012). In addition, we also analyzed the relationship between the SNPs in *CYP24A1* gene and ischemic stroke risk on the basis of stroke potential risk factors including hypertension and coronary disease (Table 6). Our results indicated that the genotype of rs1570669 was correlated with decreased risk of ischemic stroke patients with hypertension (AA genotype, OR = 0.58, 95% CI = 0.34–0.99, *p* = .048). There was no significant correlation between SNPs in the *CYP24A1* gene and risk of ischemic stroke patients with coronary disease (*p* > .05).

**Table 4:** The SNPs of *CYP24A1* gene association with ischemic stroke risk in age status

SNP	Genotype	>64		≤64	
		OR (95% CI)	*p*	OR (95% CI)	*p*
rs2762934	GG	1.00		1.00	
	AG	0.79 (0.47–1.32)	.367	1.62 (1.04–2.52)	**.033**
	AA	0.16 (0.02–1.51)	.111	1.61 (0.23–11.15)	.630
	AG–AA	0.72 (0.44–1.19)	.200	1.62 (1.05–2.51)	**.031**
rs1570669	GG	1.00		1.00	
	AG	0.63 (0.40–0.99)	**.044**	0.61 (0.41–0.91)	**.015**
	AA	0.71 (0.40–1.27)	.249	1.23 (0.72–2.10)	.453
	AG–AA	0.66 (0.43–0.99)	**.044**	0.74 (0.51–1.07)	.107
rs6068816	CC	1.00		1.00	
	TC	1.64 (1.06–2.53)	**.028**	1.05 (0.72–1.54)	.798
	TT	0.96 (0.49–1.89)	.918	1.21 (0.65–2.28)	.547
	TC–TT	1.47 (0.97–2.23)	.068	1.18 (0.65–2.16)	.582
rs2296241	GG	1.00		1.00	
	AG	0.82 (0.52–1.29)	.381	0.74 (0.48–1.13)	.158
	AA	0.65 (0.35–1.20)	.17	1.10 (0.66–1.84)	.721
	AG–AA	0.77 (0.50–1.19)	.246	0.83 (0.56–1.24)	.365

Note

*p* values were calculated by unconditional logistic regression adjusted by gender and age; *p* < .05 indicates statistical significance.

Abbreviations: CI, confidence interval; OR, odds ratio; SNP, single‐nucleotide polymorphism.

**Table 5:** The SNPs of *CYP24A1* gene association with ischemic stroke risk under stratification analysis of gender

SNP	Genotype	Female		Male	
		OR (95% CI)	*P*	OR (95% CI)	*p*
rs2762934	GG	1.00		1.00	
	AG	1.48 (0.86–2.55)	.159	1.04 (0.71–1.54)	.829
	AA	—	.999	0.80 (0.21–3.00)	.737
	AG–AA	1.38 (0.481–2.36)	.240	1.03 (0.70–1.50)	.897
rs1570669	GG	1.00		1.00	
	AG	0.46 (0.28–0.74)	**.002**	0.69 (0.49–0.97)	**.033**
	AA	1.08 (0.57–2.05)	.821	0.84 (0.53–1.32)	.447
	AG–AA	0.58 (0.37–0.91)	**.018**	0.73 (0.53–1.00)	**.049**
rs6068816	CC	1.00		1.00	
	TC	1.28 (0.80–2.03)	.307	1.28 (0.92–1.78)	.151
	TT	0.82 (0.41–1.65)	.576	1.34 (0.77–2.33)	.302
	TC–TT	1.53 (0.74–1.79)	.524	1.29 (0.94–1.77)	.122
rs2296241	GG	1.00		1.00	
	AG	1.13 (0.68–1.88)	.645	0.63 (0.44–0.90)	**.012**
	AA	0.63 (0.33–1.20)	.156	1.02 (0.65–1.62)	.927
	AG–AA	0.96 (0.59–1.56)	.868	0.72 (0.51–1.01)	.059

Note

*p* values were calculated by unconditional logistic regression adjusted by age and gender; *p* < .05 indicates statistical significance.

Abbreviations: CI, confidence interval; OR, odds ratio; SNP, single‐nucleotide polymorphism.

**Table 6:** The SNPs of *CYP24A1* gene association with ischemic stroke risk under stratification analysis of other risk factors

SNP	Genotype	Hypertension		Coronary disease	
		OR (95% CI)	*p*	OR (95% CI)	*p*
rs2762934	GG	1.00		1.00	
	AG	1.30 (0.79–2.16)	.306	1.42 (0.85–2.38)	.182
	AA	0.47 (0.06–3.42)	.455	—	—
	AG–AA	1.24 (0.76–2.03)	.388	1.36 (0.81–2.28)	.239
rs1570669	GG	1.00		1.00	
	AG	0.79 (0.51–1.24)	.310	0.95 (0.58–1.55)	.837
	AA	0.58 (0.34–0.99)	**.048**	0.96 (0.51–1.81)	.908
	AG–AA	0.71 (0.48–1.07)	.099	0.95 (0.61–1.49)	.836
rs6068816	CC	1.00		1.00	
	TC	1.02 (0.66–1.56)	.939	1.26 (0.78–2.03)	.347
	TT	0.90 (0.46–1.77)	.769	1.02 (0.47–2.20)	.965
	TC–TT	1.00 (0.66–1.50)	.982	1.21 (0.76–1.93)	.414
rs2296241	GG	1.00		1.00	
	AG	1.02 (0.65–1.62)	.918	0.87 (0.53–1.43)	.586
	AA	0.88 (0.50–1.55)	.660	0.71 (0.37–1.37)	.307
	AG‐/A	0.98 (0.64–1.50)	.923	0.82 (0.52–1.31)	.414

Note

*p* values were calculated by unconditional logistic regression adjusted by age and gender; *p* < .05 indicates statistical significance.

Abbreviations: CI, confidence interval; OR, odds ratio; SNP, single‐nucleotide polymorphism.

## 4 DISCUSSION

We firstly carried out the association analysis between four SNPs in *CYP24A1* gene and ischemic stroke susceptibility in the Chinese population. Notability, *CYP24A1* polymorphisms (rs1570669, rs2296241, rs2762934, and rs6068816) associated with ischemic stroke risk. Interestingly, *CYP24A1* polymorphisms (rs1570669 and rs2296241) played a protective role in risk of ischemic stroke, especially for the patients with hypertension. Moreover, *CYP24A1* rs6068816 polymorphism was significantly associated with an increased ischemic stroke risk in age >64 years. *CYP24A1* rs2762934 polymorphism had an enhanced susceptibility to ischemic stroke in age ≤64 years. Our results might give a new insight on relationship between *CYP24A1* polymorphisms and ischemic stroke risk.

*CYP24A1* is located on chromosome 20q13.2‐q13.3 belongs to cytochrome P450 gene family, which is a kind of vitamin D 24‐hydroxylase or 1, 25‐dihydroxyvitamin D‐24‐hydroxylase. And those two enzymes encoded by *CYP24A1* gene can catalyze 25‐OH‐D_3_ or 1α‐25‐(OH)_2_D_3_ to 24‐hydroxylated vitamin D products (Jones et al., 2014). Vitamin D is a steroid hormone, which mainly consists of two forms including 25 hydroxyvitamin D (25‐OH‐D_3_) and 1,25‐dihydroxyvitamin D (1α‐25‐(OH)_2_D_3_; Blunt et al., 1968). An increased evidences have proved that the deficiency of vitamin D is closely related to human diseases, such as colon cancer (Jacobs et al., 2013), breast or prostate cancer (McCullough, Bostick, & Mayo, 2009; Osanai & Lee, 2016), hypertension (Varakantham, Ale, Sailoo, Nagalla, & Bharatraj, 2019), coronary artery disease (Shen et al., 2010), cardiovascular health (Schwarz, Nicholls, & Psaltis, 2018), myocardial infarction, and acute stroke (Poole, Loveridge, et al., 2006). Ozben B showed that vitamin D deficiency is related to increase hypertension in healthy individuals, thus they proposed polymorphic enzyme such as *CYP24A1* might improve the degradation of active vitamin D and vitamin D deficiency would enhance the risk of stroke development complicating with hypertension (Tarcin et al., 2009). Furthermore, Varakantham et al suggested the SNPs of *CYP24A1* gene play a crucial function in disease susceptibility according to influencing the levels of 25(OH)D_3_ (Cappellani et al., 2019; Varakantham et al., 2019). Many studies revealed that genetic variants in *CYP24A1* gene have previously been investigated in relation to risk of human diseases, such as coronary artery disease (Shen et al., 2010), hypertension (Varakantham et al., 2019), and obesity with ischemic stroke (Turkanoglu Ozcelik et al., 2018) It can be seen *CYP24A1* polymorphisms association with ischemic stroke susceptibility.

In this case–control study, we focused on investigating the relationship of rs2762934, rs1570669, rs6068816, and rs2296241 in *CYP24A1* with ischemic stroke risk. Our research indicated that those four SNPs of *CYP24A1* gene played an important role in ischemic stroke risk evaluation. The rs1570669 was significantly associated with reducing susceptibility in ischemic stroke patients. To the best of our knowledge, there have not reported about correlation between rs1570669 and stroke risk. In addition, we also examined the correlation between SNPs of *CYP24A1* gene and ischemic stroke risk via stratification analysis including age, gender, hypertension, and coronary disease. Upon age‐based stratification, we found that the TC genotype of rs6068816 was associated with an increased risk of ischemic stroke, while the AG and AG–AA genotypes of rs1570669 played protective role in ischemic stroke risk of the subjects >64 years. And the AG and AG–AA genotypes of rs2762934 were a higher risk of ischemic stroke, but the AG genotype of rs1570669 could reduce the ischemic stroke risk in the subjects ≤64 years old. It seems that age is a significant factor in association between *CYP24A1* polymorphisms and ischemic stroke risk. And CT of *CYP24A1* rs6068816 polymorphisms was important in disease risk assessment, Xu et al. also revealed that the CT genotype of *CYP24A1* rs6068816 is related to an increased risk of lung cancer in Chinese female nonsmokers (Qu et al., 2019), which indicates that the same polymorphism may involve in variant human diseases.

In the gender‐stratified groups, the AG and AG–AA genotypes of rs1570669 decreased the ischemic stroke risk in both female and male subjects. And the AG genotype of rs2296241 depressed the risk of ischemic stroke in male individuals. These results suggested that gender played a risk factor role in association with *CYP24A1* variants and ischemic stroke progression. Besides, the AA genotype of rs1570669 presented a defended role in ischemic stroke susceptibility on the basis of hypertension stratification. We have not found association between *CYP24A1* polymorphisms and risk of ischemic stroke patients with coronary disease, and there were no reports about this case at present. We guess some other polymorphisms of the *CYP24A1* gene or other genes may relate to ischemic stroke with coronary disease, and it is need for further research.

Some limitations of this study are as followings. Firstly, we just identified four polymorphisms of the *CYP24A1* gene correlated with ischemic stroke risk, and more polymorphisms in *CYP24A1* associated with ischemic stroke risk are needed to detect. Secondly, the mechanism of *CYP24A1* genetic polymorphisms influenced on ischemic stroke have not investigated in our present study, future work is required to focus on this issue. Thirdly, the correlation of genetic polymorphism with hemorrhagic stroke is needed to be tested in next step. In spite of the above limitations, our present results provided a scientific foundation in association with *CYP24A1* gene and risk assessment for future research.

## CONFLICT OF INTEREST

The authors declare that they have no competing interests.

## AUTHOR CONTRIBUTIONS

YJH and WY conceived and designed the experiments. FHM, JWZ, and YHW recruited and collected study samples. TBJ selected the SNPs and designed primers. WY, HXZ, and DYY performed and analyzed the data. WY wrote the paper and YJH edited the manuscript. All authors read and approved the final manuscript.

## Data Availability

The data that support the findings of this study are available from the corresponding author upon reasonable request.
